# Recurrence of Symptoms Following a 2-Day Symptom Free Period in Patients With COVID-19

**DOI:** 10.1001/jamanetworkopen.2022.38867

**Published:** 2022-10-27

**Authors:** Davey M. Smith, Jonathan Z. Li, Carlee Moser, Eunice Yeh, Judith S. Currier, Kara W. Chew, Michael D. Hughes

**Affiliations:** 1 Department of Medicine, University of California, San Diego, La Jolla; 2 Department of Medicine, Brigham and Women’s Hospital, Harvard Medical School, Boston, Massachusetts; 3 Center for Biostatistics in AIDS Research, Harvard T.H. Chan School of Public Health, Boston, Massachusetts; 4 Department of Medicine, David Geffen School of Medicine at University of California, Los Angeles; 5 Center for Biostatistics in AIDS Research, Department of Biostatistics, Harvard T.H. Chan School of Public Health, Boston, Massachusetts

## Abstract

This cohort study of US adults with untreated COVID-19 examines the types and length of symptoms experienced following symptom recurrence.

## Introduction

Recurrence of symptoms after finishing treatment for COVID-19 with nirmatrelvir-ritonavir (Paxlovid) has become increasingly recognized.^1,2,3^ The biological underpinning of this phenomenon is unclear, and its etiology may be multifactorial, including rapid clearance of nirmatrelvir coupled with delayed immune responses or possible development of drug resistance.^1,2,3^ The contribution of treatment to symptom rebound needs to be differentiated from symptom rebound that might occur during the natural history of COVID-19. In this cohort study, we sought to determine how often COVID-19 symptoms recurred when the disease was untreated.

## Methods

We assessed COVID-19 symptoms for 29 days in untreated participants who received a placebo in the ACTIV-2/A5401 trial between August and November 2020, following Strengthening the Reporting of Observational Studies in Epidemiology (STROBE) guidelines for cohort studies. ACTIV-2/A5401 (NCT04518410) is a phase 2/3 platform trial to evaluate the safety and efficacy of investigational agents to treat nonhospitalized adults (aged 18 years and older) with COVID-19.^4^ The Advarra institutional review board approved the study; all participants provided written informed consent. Participants had documented SARS-CoV-2 infection and 10 or fewer days experiencing COVID-19 symptoms at study entry.^4^ Participants completed a daily symptom diary from enrollment (day 0) to day 28, which included 13 COVID-19 symptoms, scored by the participant as absent, mild, moderate, or severe (Figure).^4^ We assessed the frequency of targeted symptom recurrence after symptom resolution, defined as all 13 symptoms reported absent for 2 consecutive days.

**Figure.**
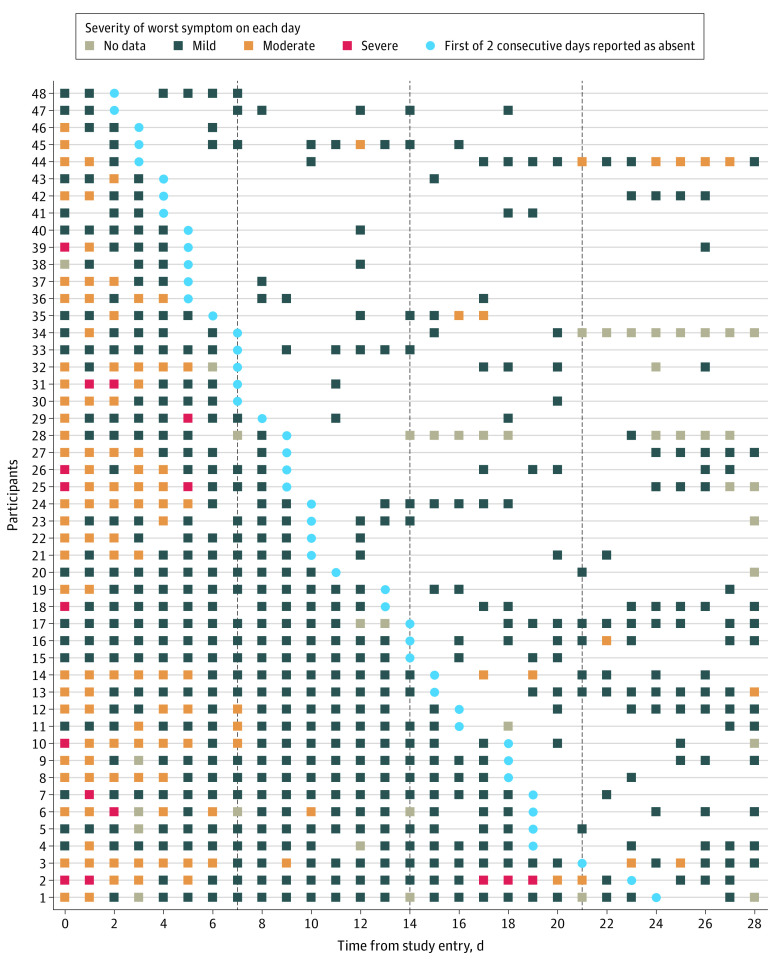
Self-reported Symptoms and Their Severity, Experienced by Participants Who Had At Least 2 Consecutive Days with All Targeted Symptoms Absent Boxes indicate each day on which participants recorded in their diary at least 1 of 13 targeted symptoms being present at a self-reported severity of mild, moderate, or severe. The color of the box reflects the worst symptom severity reported of all symptoms recorded as being present. No participants reported severe symptoms after being symptom free for 2 days. Most recurrent symptoms were self-reported as mild.

## Results

Among the 158 evaluable participants, 79 (50%) were women, median (IQR) age was 47 years (34-55 years), 28 (18%) self-identified as a minoritized racial group and 48 (31%) as having Hispanic ethnicity. Median (IQR) time from symptom onset to enrollment was 6 days (4-7 days). Sixty-six (42%) reported a high-risk condition, with the highest frequency conditions being hypertension (44 participants [28%]) and obesity (ie, body mass index [calculated as weight in kilograms divided by height in meters squared] greater than 35) (17 participants [11%]).^4^

During 28 days of follow-up, 108 participants (68%) achieved symptom resolution, of which 48 (44%; 30% of the 158 participants) subsequently reported recurrence by the end of the 28 days of follow-up of at least 1 of the 13 targeted symptoms (Table). Among those reporting recurrence of symptoms after reporting resolution of symptoms for 2 consecutive days, 41 participants (85%) reported their symptoms as mild, 7 (15%) reported at least 1 moderate symptom and none reported severe symptoms during recurrence. The most common symptoms reported at time of relapse were cough (21 participants [44%]), fatigue (17 participants [35%]), and headache (17 participants [35%]); these results were similar to symptoms at enrollment, with the exception that body pain and aches were reported more often at enrollment than on recurrence (Table). This profile of symptoms on recurrence was like the profile of persistent symptoms at days 27 and 28 for participants who never resolved all symptoms at end of follow-up (data not shown). Eight hospitalizations (5%) but no deaths occurred among the 158 participants, none occurring among participants who achieved symptom resolution and then experienced recurrence.

**Table.** Symptoms Reported on First Day of Recurrence After at Least 2 Consecutive Days with All Targeted Symptoms Reported as Absent

Symptoms	Participants with symptom reoccurrence, No. (%)		
	Mild	Moderate	Total
Reporting at least 1 symptom	41 (85)	7 (15)	48 (100)
Cough	19 (40)	2 (4)	21 (44)
Fatigue	16 (33)	1 (2)	17 (35)
Headache	15 (31)	2 (4)	17 (35)
Nasal obstruction or congestion	13 (27)	1 (2)	14 (29)
Sore throat	10 (21)	NA	10 (21)
Nasal discharge	6 (13)	1 (2)	7 (15)
Body/muscle pain	6 (13)	1 (2)	7 (15)
Shortness of breath	5 (10)	NA	5 (10)
Chills	3 (6)	NA	3 (6)
Feverish	3 (6)	NA	3 (6)
Nausea	2 (4)	NA	2 (4)
Diarrhea	2 (4)	NA	2 (4)
Vomiting	1 (2)	NA	1 (2)

Abbreviation: NA, not applicable.

## Discussion

Using daily symptoms data from a prospective trial, we found the natural history of untreated COVID-19 was variable and undulating. Over one-third of participants who experienced symptom resolution for at least 2 consecutive days within the first 4 to 5 weeks of COVID-19 symptoms reported recurrent symptoms. Reported symptoms are inherently subjective, and our observed variation may explain some of the rebound of symptoms after treatment for COVID-19, like in cases of what has been described as Paxlovid rebound.^1,2,3^

This study was limited by lack of virologic characterization, including the presence of simultaneous viral rebound. Also, participants were enrolled before COVID-19 vaccinations were available and when the original and Alpha variant SARS-CoV-2 strains circulated, so these results may not be generalizable to the current pandemic, where Omicron variants predominate.^5^ Consistent with an earlier report,^6^ our results in persons with untreated COVID-19 shows that recurring symptoms are common among those who initially improve, but these recrudescent symptoms do not portend progression to severe COVID-19.

## Notes

Supplement.
Nonauthor Collaborators
