# Supplementary material for: Recurrence of Symptoms Following a 2-Day Symptom Free Period in Patients With COVID-19
Source: JAMA Netw Open. 2022 Oct 27;5(10):e2238867. doi: 10.1001/jamanetworkopen.2022.38867 (PMC9614575; doi:10.1001/jamanetworkopen.2022.38867)
Supplement: Supplement. — Nonauthor Collaborators [file jamanetwopen-e2238867-s001.pdf]

*First name, last name, and suffix (if applicable) are required and will appear in PubMed.

***Group Name(s): ACTIV-2/A5401 Study Team**

*First Name and Middle Initial(s)	*Last Name	*Suffix (eg, Jr, III)	Academic Degrees	Institution	Location (city, state/province, country)	Role or Contribution, eg, chair, principal investigator	Group (if more than 1 Group listed in the byline) and/or Subgroup (eg, Steering Committee)
Eric	Daar		MD	Lundquist Institute at Harbor-UCLA Medical Center	Torrance, CA, USA	Vice Chair	
David	Wohl		MD	University of North Carolina at Chapel Hill School of Medicine	Chapel Hill NC, USA	Vice Chair	
Joseph	Eron		MD	University of North Carolina at Chapel Hill School of Medicine	Chapel Hill NC, USA	Protocol Investigator	
Arzhang C.	Javan		MD, MPH, DTM&H	NIH Division of AIDS (DAIDS) Clinical Representative, National Institutes of Health	Rockville, MD, USA	Medical Officer	
Mark	Giganti		PhD	Harvard T.H. Chan School of Public Health	Boston, MA, USA	Statistician	
Justin	Ritz		MS	Harvard T.H. Chan School of Public Health	Boston, MA, USA	Statistician	
Lara	Hosey		MA	AIDS Clinical Trials Group (ACTG) Network Coordinating Center, Social & Scientific Systems, a DLH Company	Silver Spring, MD, USA	Clinical Trials Specialist	
Jhoanna	Roa		MD	AIDS Clinical Trials Group (ACTG) Network Coordinating Center, Social & Scientific Systems, a DLH Company	Silver Spring, MD, USA	Clinical Trials Specialist	
Nilam	Patel			AIDS Clinical Trials Group (ACTG) Network Coordinating Center, Social & Scientific Systems, a DLH Company	Silver Spring, MD, USA	Clinical Trials Specialist	
Kelly	Colsh		PharmD	NIH/DAIDS Pharmaceutical Affairs Branch	Rockville, MD, USA	DAIDS Pharmacist	
Irene	Rwakazina		PharmD	NIH/DAIDS Pharmaceutical Affairs Branch	Rockville, MD, USA	DAIDS Pharmacist	
Justine	Beck		PharmD	NIH/DAIDS Pharmaceutical Affairs Branch	Rockville, MD, USA	DAIDS Pharmacist	
Scott	Seig		PhD	Case Western Reserve University	Cleveland, OH, USA	Protocol Immunologist	
Courtney	Fletcher		PharmD	University of Nebraska Medical Center	Omaha, NE, USA	Protocol Pharmacologist	
William	Fischer		MD	University of North Carolina at Chapel Hill School of Medicine	Chapel Hill NC, USA	Protocol Critical Care Specialist	
Teresa	Evering		MD	Weill Cornell Medicine	New York, NY, USA	Protocol Investigator	
Rachel	Bender		MD	University of Washington	Seattle, WA	Protocol Investigator	
Sandra	Cardoso		MD, PhD	Fundação Oswaldo Cruz	Rio de Janeiro, Brazil	Protocol Investigator	
Katya	Corado		MD	Lundquist Institute at Harbor-UCLA Medical Center	Torrance, CA, USA	Protocol Investigator	
Prasanna	Jagannathan		MD	Stanford University School of Medicine	Palo Alto, CA, USA	Protocol Investigator	
Nikolaus	Jilg		MD	Massachusetts General Hospital, Harvard Medical School	Boston, MA, USA	Protocol Investigator	
Alan	Perelson		PhD	Los Alamos National Laboratory	Los Alamos, NM, USA	Protocol Investigator	
Sandy	Pillay		MB, CHB	Enhancing Care Foundation	Durban, KwaZulu-Natal,	Protocol Investigator	
Cynthia	Riviere		MD	GHESKIO Center	Port-au-Prince, Haiti	Protocol Investigator	
Upinder	Singh		MD	Stanford University School of Medicine	Palo Alto, CA, USA	Protocol Investigator	
Babafemi	Taiwo		MBBS, MD	Northwestern University Feinberg School of Medicine	Chicago, IL, USA	Protocol Investigator	
Joan	Gottesman		BSN, RN,	Vanderbilt University Medical Center	Nashville, TN, USA	Field Representative	
Matthew	Newell		BSN, RN,	University of North Carolina at Chapel Hill School of Medicine	Chapel Hill NC, USA	Field Representative	
Susan	Pederson		BSN, RN	University of North Carolina at Chapel Hill School of Medicine	Chapel Hill NC, USA	Field Representative	
Joan	Dragavon		MLM	University of Washington	Seattle, WA, USA	Laboratory Technologist	
Cheryl	Jennings		BS	Northwestern University	Chicago, IL, USA	Laboratory Technologist	
Brian	Greenfelder		BA	Ohio State University	Columbus, OH, USA	Laboratory Technologist	
William	Murtaugh		MPH	ACTG Laboratory Center, University of California, Los Angeles	Los Angeles, CA, USA	Laboratory Technologist	
Jan	Kosmyna		MIS, RN, (	Case Western University Clinical Research Site	North Royalton, OH, US/	ACTG Community Scientific Subcommittee Representative	
Morgan	Gapara		MPH	ACTG Network Coordinating Center, Social & Scientific Systems, a DLH Company	Durham, NC, USA	International Site Specialist	
Akbar	Shahkolahi		PhD	ACTG Network Coordinating Center, Social & Scientific Systems, a DLH Company	Silver Spring, MD, USA	International Site Specialist	
Peter	Kim		MD	NIH Division of AIDS (DAIDS) Clinical Representative, National Institutes of Health	Rockville, MD, USA	Investigator	
William	Erhardt		MD	NIH Division of AIDS (DAIDS) Clinical Representative, National Institutes of Health	Rockville, MD, USA	Investigator	
